# Optimization and Characterization of Candidate Strain for Coxsackievirus A16 Inactivated Vaccine

**DOI:** 10.3390/v7072803

**Published:** 2015-07-17

**Authors:** Jingliang Li, Guanchen Liu, Xin Liu, Jiaxin Yang, Junliang Chang, Wenyan Zhang, Xiao-Fang Yu

**Affiliations:** 1 First Hospital of Jilin University, Institute of Virology and AIDS Research, 130061 Changchun, China; E-Mails: jingliang_li1030@163.com (J.L.); fry88614822@163.com (G.L.); liuxin87529524@163.com (X.L.); yjx19861122@163.com (J.Y.); cjlcc2009@163.com (J.C.); 2 Department of Molecular Microbiology and Immunology, Johns Hopkins Bloomberg School of Public Health, 615 N. Wolfe Street, Baltimore, MD 21205, USA

**Keywords:** Coxsackievirus A16, HFMD, vaccine candidate strain, inactivated vaccine

## Abstract

Coxsackievirus A16 (CA16) and enterovirus 71 (EV71), both of which can cause hand, foot and mouth disease (HFMD), are responsible for large epidemics in Asian and Pacific areas. Although inactivated EV71 vaccines have completed testing in phase III clinical trials in Mainland China, CA16 vaccines are still under development. A Vero cell-based inactivated CA16 vaccine was developed by our group. Screening identified a CA16 vaccine strain (CC024) isolated from HFMD patients, which had broad cross-protective abilities and satisfied all requirements for vaccine production. Identification of the biological characteristics showed that the CA16CC024 strain had the highest titer (10^7.5^ CCID_50_/mL) in Vero cells, which would benefit the development of an EV71/CA16 divalent vaccine. A potential vaccine manufacturing process was established, including the selection of optimal time for virus harvesting, membrane for diafiltration and concentration, gel-filtration chromatography for the down-stream virus purification and virus inactivation method. Altogether, the analyses suggested that the CC-16, a limiting dilution clone of the CC024 strain, with good genetic stability, high titer and broad-spectrum immunogenicity, would be the best candidate strain for a CA16 inactivated vaccine. Therefore, our study provides valuable information for the development of a Vero cell-based CA16 or EV71-CA16 divalent inactivated vaccine.

## 1. Introduction

Coxackievirus A16 (CA16) and enterovirus 71 (EV71) are two major etiological agents of hand, foot and mouth disease (HFMD). HFMD has become a severe pubic health problem and caused both economic difficulties and social panic [1]. Since 2008, the Chinese government identified HFMD as a class C infectious disease in Mainland China [2], and the development of a HFMD vaccine was given increased attention in order to prevent and control this disease. However, no vaccine is available currently for public use. Moreover, the development of a HFMD vaccine has targeted mainly EV71 [3], since infection with this virus presents with neurological manifestations and has even resulted in some fatal cases [4,5,6]. However, recent clinical investigations showed that CA16 was responsible not only for HFMD infections, but it also was associated with severe health issues, such as aseptic meningitis, rhombencephalitis, cardiac and pericardial disease, pulmonary complications, and even lethal myocarditis [7,8,9,10,11,12]. Moreover, the co-circulation of CA16 and EV71 has resulted in co-infections by the two viruses, which may have led to their recombination [13,14] and generation of a recombinant virus responsible for a large HFMD outbreak in the Chinese mainland [15].

At present, inactivated EV71 vaccines developed in Mainland China have completed phase III clinical trials [16,17,18]. The results showed that the EV71 vaccines could provide good safety and protection against EV71-associated HFMD or herpangina in infants and young children. Recently, several types of CA16 vaccines, including inactivated CA16 and virus-like particles (VLPs) [19], have been evaluated in animals but not yet tested in a clinical trial. To ensure the broad and effective protection against HFMD, the CA16 virus should also be targeted for development, especially of an EV71 and CA16 divalent vaccine.

In this study, we screened CA16 vaccine candidate strains from a series of CA16 viruses isolated from HFMD patients according to the national standard antigen and neutralizing antibody responses for EV71 vaccines suggested by Liang *et al.* [19,20] in 2011. The vaccine candidate strain CA16CC024 is well-adapted to the Vero cell line, which is one of the most popular continuous cell lines used for manufacturing human vaccines [21,22,23]. The CA16CC024 virus formulated with aluminum hydroxide (alum) adjuvant could elicit strong CA16-specific humoral responses in mice. Sera from mice immunized with the CA16 candidate strain neutralized both homologous and heterologous CA16 clinical isolates and SHZH05 as well as the prototype G10 strain. As demonstrated previously, this candidate strain could protect neonatal mice born to immunized female mice from lethal-dose challenge with a series of CA16 viruses [22]. A manufacturing process for producing the CA16 inactivated vaccine was also established, including the selection of the optimal time for virus harvest, methods for viral inactivation and gel filtration for down-stream purification. Therefore, these results provide valuable information for development of a Vero cell-adapted CA16 inactivated vaccine.

## 2. Materials and Methods

### 2.1. Ethics Statement

This study was conducted in accordance with the Declaration of Helsinki, and the protocol was approved by the Ethics Committee at the First Hospital of Jilin University. Written informed consent was obtained from the parents of all children involved in our study. All animal experiments were approved by the Animal Care and Use Committee at the First Hospital of Jilin University.

### 2.2. Cells and CA16 Viruses

The African green monkey Vero cell line, which was obtained from the American Type Culture Collection (ATCC, cat. no. CCL-81), was grown in Eagle’s medium (MEM, supplemented with 8% fetal bovine serum (FBS)) at 37 °C with 5% CO_2_ in T25 flasks, T75 flasks or cell factories. Various throat swab virus samples were collected from patients of different HFMD epidemics. The samples were suspended in 2 mL Hank’s medium and centrifuged at 3000× *g* for 20 min. The cleared supernatant was sterilized by passing through a 0.22-µm filter and then inoculated into Vero cells in 6-well plates. Following the detection of cytopathic effects (CPEs) and identification by PCR sequences, the inoculated Vero cells were harvested by continuous passage. Thereafter, all CA16 strain stocks (prototype strain G10/U05876, Shenzhen05 strain/EU262658) were propagated on confluent Vero cell monolayers in MEM with 2% FBS. Virus characterization and titer tests were also carried out using Vero cells.

### 2.3. Determination of Viral Titer

Virus titers were determined using the median end point of the cell culture’s infectious dose (CCID_50_). Serially-diluted viruses were added to Vero cells grown in 96-well plates, and 8 replicate samples were used for each dilution. The 96-well plates were incubated for 7 days at 35 °C, and the CCID_50_ values were measured by counting infected Vero cell culture wells with obvious CPEs and calculated by the Reed–Muench method [23].

For quantitative real-time PCR (qRT-PCR), viral RNA was extracted using TRIzol reagent (Invitrogen, Carlsbad, CA, USA) from the collected samples and 10-fold serially diluted. The cDNA was generated using the High-capacity cDNA Reverse Transcription Kit (Applied Biosystems, Foster, CA, USA) and Oligo-d (T)_18_ primers according to the supplier’s instructions. Sequences of primers, designed using the VP1 conserved region sequences of CA16, were as follows: CA16-F1: CATGCAGCGCTTGTGCTT; CA16-F2: CATGCAACGACTGTGCTTTC; CA16-R1: CACACAATTCCCCCGTCTTAC; and CA16-R2: CATAATTCGCCCGTTTTGCT. The SYBR green-based real-time RT-PCR was carried out on an Mx3005P instrument (Agilent Technologies Stratagene, Santa Clara, CA, USA) using the double-stranded DNA-binding dye method with a SYBR^®^ Green PCR Master Mix (Applied Biosystems). Each 20 µL reaction mixture contained 10 µL SYBR Premix; 0.2 µL (10 µM) each of F1, R1, F2 and R2; 7.2 µL ddH_2_O; and 2 µL of cDNA templates. Cycling conditions were as follows: 50 °C for 2 min, then 95 °C for 10 min, followed by 50 cycles consisting of 95 °C for 15 s and 60 °C for 1 min. The melting curve analysis was conducted at 90 °C for 1 min, then 55 °C for 30 s and 95 °C for 30 s.

### 2.4. Characterization of CA16 Viral Particles by Electron Microscopy

The CA16 virus culture supernatant was harvested, and the cell debris was removed by centrifugation. The supernatant was concentrated 5-fold with a 50 K Amicon unit (Millipore, Billerica, MA, USA). The resultant 1.5 mL concentrate was loaded onto a 20%–60% continuous sucrose gradient and centrifuged at 28,000 rpm for 4 h using a zonal rotor of SW 41 Ti (Beckman, Brea, CA, USA). The fractions (each 0.5 mL) were collected for Western blot and infectivity analyses. The virus samples were subjected to negative staining with 0.5% aqueous uranyl acetate, and transmission electron microscopy was performed with a Philips CM-12S microscope.

### 2.5. Neutralization Assay

Neutralization titers were determined by the CCID_50_ reduction assay in Vero cells. Serum samples were 2-fold serially diluted with MEM and heat-inactivated at 56 °C for 30 min, while the CA16 stock was diluted to a working concentration of 200 CCID_50_. Thereafter, 50 μL of each diluted serum sample was mixed with 50 μL of CA16 virus and added to 96-well plates for incubation at 37 °C for 2 h. Following the incubation, 100 μL of a Vero cell suspension (2 × 10^5^ cell/mL) was seeded into each well of the 96-well plates for infection and cultured at 35 °C with 5% CO_2_. At 7 days post-infection, the cells were observed under a microscope for the presence of CPEs. Neutralization titers were determined as the highest serum dilution that could prevent CPE in >50% of cells.

### 2.6. Viral Genome Sequencing

Viral RNA was extracted from 200 µL of different harvested viruses using Trizol reagent (Invitrogen). The cDNA was generated using a Reverse Transcription Kit (Applied Biosystems) and oligo-dT primers according to the supplier’s instructions. PCR parameters for all primer pairs were as follows: cDNA were denatured at 94 °C for 5 min. The amplification was performed in 35 cycles consisting of a denaturing step for 30 s at 94 °C, a primer annealing step for 30 s at 50 to 55 °C, followed by a two-part elongation step for 1 to 2 min at 72 °C and then extension at 72 °C for 8 to 10 min. Amplifications were either sequenced directly by Sangon Biotech (Shanghai Co. Ltd., Shanghai, China) using the BigDye terminator v3.1 kit and ABI-PRISM3730XL DNA sequencer (Applied Biosystems). The CA16 full-length genomes were acquired by assembling all of the fragments using the DNAMAN5.2.2 software (LynnonBiosoft, San Ramon, CA, USA).

### 2.7. Viral Growth Assay

Confluent Vero cells were infected with CA16 virus. For determining the replication kinetics of various CA16 and CC024 clone strains, Vero cells were infected at the multiplicity of infection (MOI) of 0.01 at 35 °C. For evaluating replication kinetics of CA16, Vero cells were infected at 0.0001, 0.001, 0.01 and 0.1 MOI at 35 °C. The infected Vero samples were collected at different time points, and then titers of all collected samples were tested. The different MOI and time points were determined by CCID_50_, and the titer values were used for plotting growth curves.

### 2.8. Pilot Production of CA16 Vaccine

Vero cells were grown in 10-layer cell factories (Nunc Thermo Fisher Scientific) in 2 L of serum-containing medium (Eagle’s MEM plus 8% fetal bovine serum). When the Vero cells had formed tight monolayers, they were infected with the Working Cell Bank (WCB) CC-16 virus at the MOI of 0.01 and cultured in 35 °C. At 4 days post-infection, the culture supernatant of each cell factory was collected and combined. The cell debris was removed by using a Zeta Plus filter (3 M, Saint Paul, Minnesota, USA). The crude virus bulk was concentrated 20- to 30-fold with a 100 KDa cut-off diafiltration membrane in a TFF capsule (Millipore, USA) and then purified using an AKTA Pilot liquid chromatography system purchased from GE Healthcare (USA) equipped with Sepharose 6 Fast Flow gel. Phosphate buffered solution (PBS) was used as the eluting buffer, and the flow rate was set at 9.0 mL/min. Fractions were collected and analyzed by Western blotting. Fractions containing the virus were pooled, further concentrated and then inactivated with 1:4000 β-propionolactone (BPL) at 4 °C for 72 h. Titration of samples collected at different times demonstrated that the efficiency of virus inactivation by BPL was 100% after 72 h. The vaccine bulk was obtained after sterile filtration using a 0.22-µm filter, analyzed for protein concentration by the BCA protein assay and subjected to silver staining and Western blotting as described above.

### 2.9. Mouse Immunization

The inactivated virus protein was mixed with aluminum hydroxide phosphate (final concentration 0.8 mg/mL) at room temperature for 8 h. Each group of female ICR mice at the age of 6–8 weeks were intraperitoneally (i.p.) immunized with 0.5 mL inactivated CA16 immunogens, and the mice were boosted with the same dose three weeks after priming. Blood samples were collected before immunization and 1 week after the boost and then stored at −80 °C for subsequent immunological analysis. The specificity and titer of the CA16 antisera were tested by an antibody neutralization assay.

### 2.10. Western Blot

The virus samples were lysed in 1× loading buffer (0.08 M Tris, pH 6.8, with 2.0% SDS, 10% glycerol, 0.1 M dithiothreitol, and 0.2% bromophenol blue) and boiled for 5 min, and proteins were separated by SDS-PAGE. The membranes were probed with various primary antibodies against the proteins of interest. The secondary antibodies were alkaline phosphatase-conjugated anti-mouse (Jackson Immunoresearch, Suffolk, UK) antibodies, and staining was carried out with 5-bromo-4-chloro-3-indolyl phosphate (BCIP) and nitroblue tetrazolium (NBT) solutions prepared from chemicals obtained from Sigma (Saint Louis, Missouri, USA).

### 2.11. Statistical Analysis

Values for neutralizing antibody titers and viral titers were analyzed using GraphPad Prism 6 software (GraphPad Software, La Jolla, CA, USA). Results are expressed as means ± standard error of mean (SEM). *p* < 0.05 was considered significant.

## 3. Results

### 3.1. Isolation and Characterization of CA16CC024 Vaccine Strain

**Figure 1:**
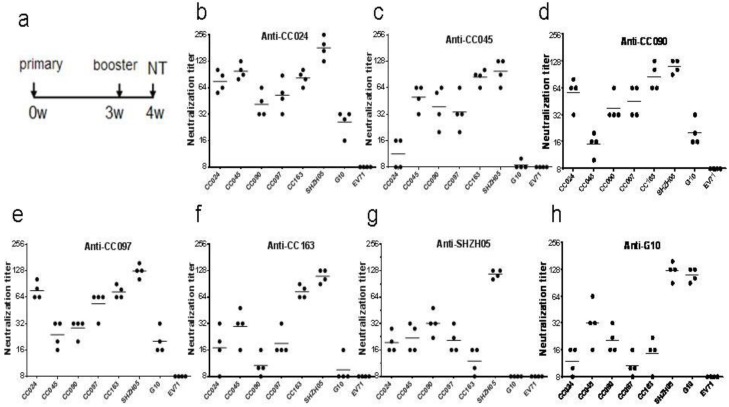
Cross-neutralization assay of various CA16 isolated strains, SHZH05 and G10. (**a**) Immunization schedule for all virus strains; (**b**–**h**) Serum neutralization titers of CC024, CC045, CC090, CC097, CC163, SHZH05 and G10 against other CA16 and EV71, respectively. Neutralization titers (Y-axis) are plotted on a logarithmic scale.

In a previous study, we reported on the biological characteristics of a series of CA16 strains isolated from different patients with mild or severe symptoms in the First Hospital of Jilin University. They were genetically distinct from the prototype CA16 G10 strain and determined to be recombinant viruses involving multiple type A human enteroviruses (HEV), including CA4, CA16 G10 and EV71A [24]. In order to develop a CA16 vaccine and satisfy the standards for innovative vaccine development according to EV71 vaccine [20], we isolated 12 CA16 viruses from hospitalized patients diagnosed with CA16 infection by use of an RT-PCR detection kit. Based on their replication kinetics and adaptation in Vero cells, five strains were selected for further analysis of immunogenicity in mice. These viruses were grown in Vero cells in T-flasks with MEM medium containing 2% FBS and harvested on the fourth day post-infection. On a research scale, these viruses were purified by 20% sucrose gradient ultracentrifugation and inactivated at 56 °C for 30 min. In order to test and compare the neutralization efficiency against other CA16 CC viruses, G10 prototype strain or SHZH05 strain, eight groups of eight mice each were immunized i.p. twice with a three-week interval with 0.5 mL of inactivated virus at 5 µg per dose (Figure 1A). Sera collected from mice immunized with the CA16 CC024 strain at one week after the booster immunization were found to be more antigenic, showing cross-reactivity and neutralizing activity against various CA16 viruses, than other CA16 strains (Figure 1). They showed high neutralization titers ranging from 56 to 436 against CC024, CC045, CC090, CC097, CC163 and SHZH05, while the neutralization titers against G10 ranged from 16 to 32. Surprisingly, none of the seven anti-sera obtained from mice immunized with various CA16 viruses formulated with aluminum hydroxide could neutralize EV71 viruses, which showed that the CA16 vaccine could not substitute for the EV71 vaccine.

### 3.2. Adaptation of CA16 Strains to Vero Cells

**Figure 2:**
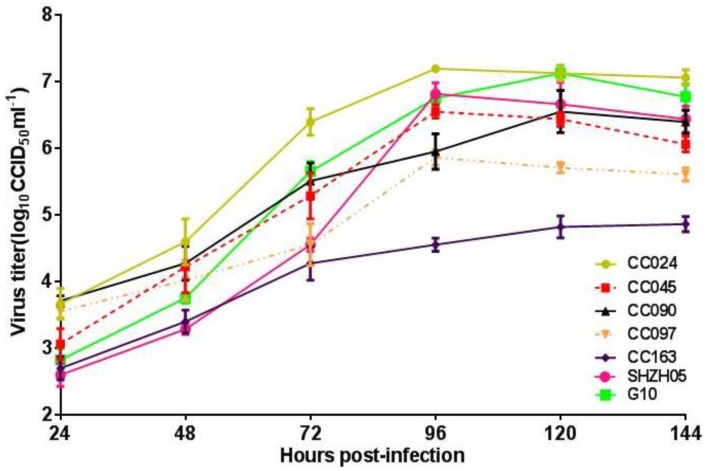
Growth kinetics of various CA16 virus strains in Vero cells. Cells were infected at the MOI of 0.01, and the titer of the CC024 strain (10^7.2^ CCID_50_/mL) was the highest among the seven viruses at 96 h post-infection

To confirm the candidate strain was adapted to grow in Vero cells by serial passage, we detected the infectious titer of CA16CC024 and other strains including SHZH05 and G10 to Vero cells. Various CA16 viruses at a MOI of 0.01 were used to infect Vero cells in T25 flasks, and their virus growth profiles were compared. At the time points of 24, 48, 72, 96, 120 and 144 h, the viruses were harvested for titer determination. At 96 h, CA16CC024 reached the highest titer of 10^7.2^ CCID_50_/mL on the fourth day post-infection when compared with other CA16 strains (Figure 2). These results suggested that CA16CC024 could induce extensive neutralizing antibodies and also showed good adaptability to Vero cells. Therefore, CA16CC024 was selected as a candidate vaccine strain for further study.

### 3.3. Biological Characterization of CA16CC024 Vaccine Strain

**Figure 3:**
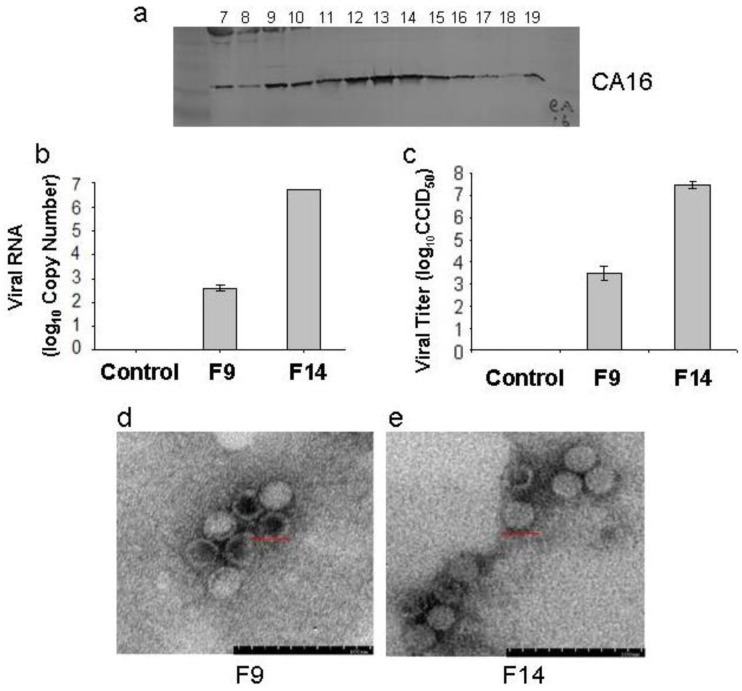
Biological characteristics of CC024 vaccine strain. (**a**) Concentrated virus was layered onto 20%–60% sucrose gradients and subjected to ultracentrifugation as described in Materials and Methods. Nineteen fractions were taken from the top to bottom and assayed. The odd fractions were analyzed by Western blot using a CA16 polyclonal antibody; (**b**) Viral RNAs of peaks at F9 and F14 were detected by RT-PCR; (**c**) Viral titers of peaks at F9 and F14 were detected in Vero cell lines; (**d**,**e**) Electron microscopy of CC024 virus from peaks at F9 and F14. Bar = 200 nm.

To better understand its biological characteristics, we performed electron microscopy and viral structural analysis on the CC024 strain. The supernatant from Vero cells infected by CC024 was harvested, concentrated and then layered onto 20%–60% sucrose gradients. The resultant fractions were assayed for the presence of capsid subunit proteins. Western blotting analysis with a CA16 whole virus-specific polyclonal antibody (made in our laboratory) revealed that the majority of virus accumulated in two peaks; one was from Fraction 9 to 10, and another peak was from Fraction 12 to15 (Figure 3a). To further investigate the difference between the two peaks, we detected the viral RNA by RT-PCR (Figure 3b) and viral titer by determining the CCID_50_ (Figure 3c)_._ The infectious virus RNA with the higher titer was found in Fraction 14, while the infectious virus RNA with the lower titer was found in Fraction 9.

In this study, the physical structure of the CA16 viral particles in Fractions 9 and 14 were observed by transmission electron microscopy. As shown in Figure 3d, we saw two types of viruses, empty/defective viral particles (hollow-core particles) and infectious viral particles (solid-core particles). More hollow-core particles than solid-core particles were seen in Fraction 9, while more solid-core particles than hollow-core particles were found in Fraction 14. These observations may explain the lower viral titer in Fraction 9 compared with that in Fraction 14. The *Picornaviradae* pre-virion consists of the P1 polypeptide precursor, which is specifically cleaved into VP0, VP1 and VP3 proteins by the viral nonstructural protein, 3CD protease. The final infective particle is assembled when the VP0 protein is then cleaved into VP2 and VP4 by an autocatalytic action that involves viral RNA [25,26,27].

### 3.4. Cloning and Identification of CA16CC024 in Vero Cells

**Figure 4:**
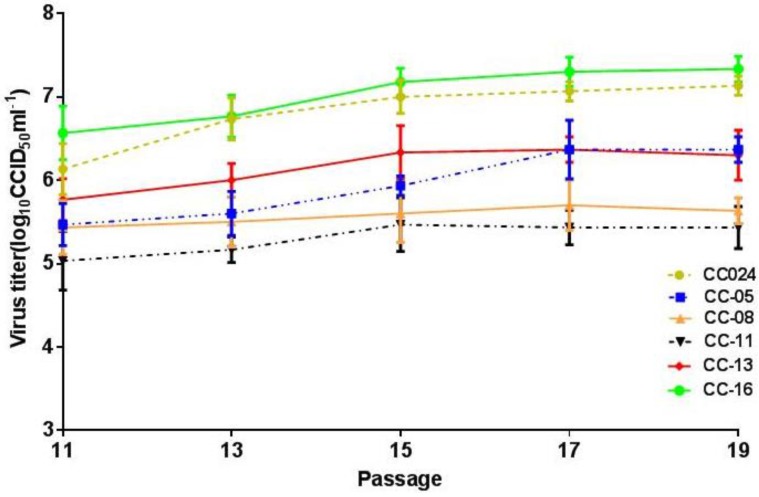
Growth curves of CC024, CC-05, CC-08, CC-11, CC-13 and CC-16 strains during passage in Vero cells infected at the MOI of 0.01. The titer of the CC-16 strain (10^7.2^CCID_50_/mL) was the highest at 96 h post-infection.

To screen for a CA16 clone with rapid growth and high yield, terminal dilution analysis was performed to obtain pure clones of the CC024 strain in Vero cells. The virus clones prepared from CA16 virus seed stocks underwent further evaluation, including genetic characterization, growth curve analysis and pilot production in cell factories. The virus clones and parent viruses were compared by growth curves in Vero cells at the MOI of 0.01 (Figure 4), which showed that the titer of the CC-16 clone was the highest. Our previous studies demonstrated that CC024 is a complex recombinant that differs from the prototype G10 in most regions of the viral genome. The 5′-UTR of the CC024 sequence is relatively similar to CA4, while its 3′ half has some similarity to EV71. Those results also indicated a possible recombination in the P2 and P3 regions between CA16 and a virus that is related to EV71A, CA2, CA3, CA6, CA10 or CA12 [24]; however, the P1 region is more similar to G10. Thus, recombination with other prevalent enteroviruses may have resulted in several amino acid changes [28,29].

**Figure 5:**
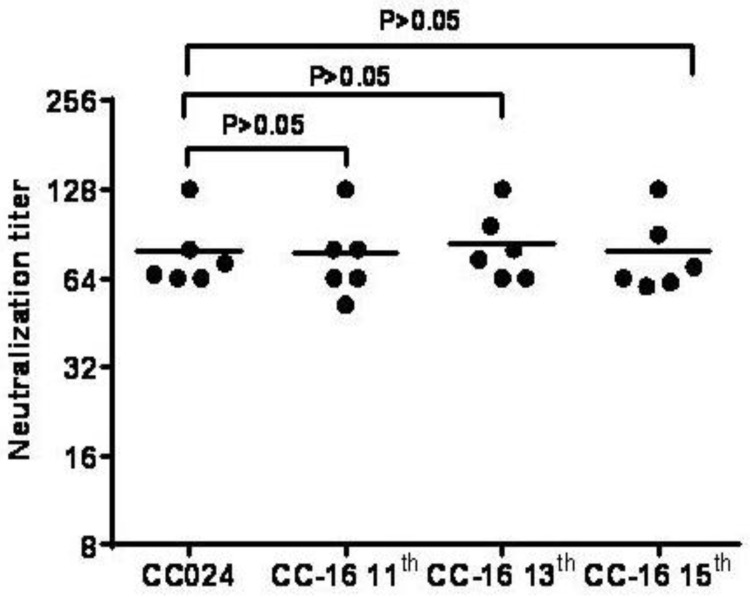
Neutralizing antibody titers of the 11th, 13th and 15th passages of CA16 CC-16. ICR mice (*n* = 6) were immunized with the same procedure and dose. Neutralization titers of the sera were determined as described in Materials and Methods and plotted (Y-axis) on a logarithmic scale.

During further adaptation of the CC-16 strain in Vero cells, the 11th, 13th and 15th passages of the CC-16 clone were determined to be the primary, master and working seeds, respectively, for vaccine production. In order to investigate their genetic stability, we sequenced the VP1 region of different passages. The amino acid sequences of the 11th, 13th and 15th passages were identical in the VP1 region, which is the main structural protein of the virus with antigenic properties, and it differed in strains CC024 and CC-16 by only one nucleotide substitution: G to T at the 397th position, which did not induce an amino acid change. No significant variation was seen in VP1, VP2 and VP3 structural protein genes related to viral immunogenicity of the CC-16 strain from the primary, master and working seed passages, indicating genetic stability and maintenance of immunogenicity (Table 1). Only one amino acid change was observed at position 51 (Arg to Lys) in the VP4 region, which is positioned in the interior of the structural protein of the CA16 virus and cannot alter the global structure of the viral capsid (Table 1). These results confirmed that this clone possessed the genetic stability required for a candidate vaccine strain. Finally, the CC-16 clone was identified as the best candidate strain for development of a CA16 inactivated vaccine. Analysis of the anti-sera from mice inoculated with the 11th, 13th and 15th passages of the CC-16 virus showed similar neutralization titers against the CC-16 clone (Figure 5).

**Table 1:** Genetic stability of CC-16 strain at different passages in Vero cells.

Characteristic		CC024	CC-16 Passage		
			11th	13th	15th
Genetic Homology	VP1	-	-	-	-
	VP2	-	-	-	-
	VP3	-	-	-	-
	VP4	Arg(51)	Lys	Lys	Lys

### 3.5. Preliminary Upstream Process Development

**Figure 6:**
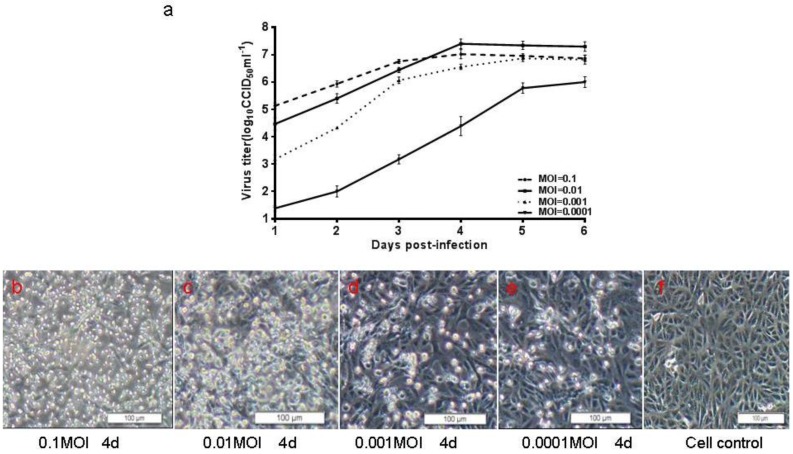
Growth kinetics of CC-16 strain and images of infected Vero cells. (**a**) Confluent Vero cells in cell factories were infected with CA16 at the MOI of 0.0001, 0.001, 0.01 and 0.1. The highest titer was 10^7.5^ CCID_50_/mL achieved with the MOI of 0.01; (**b**–**f**) Images of Vero cells infected with CC-16 on the fourth day post-infection; (**g**) Image of control Vero cells. Bar = 100 µm.

The adaptation of the CA16 CC-16 strain to Vero cells revealed that the infectious titer reached the peak in shortened time during continuous passaging (Figure 4). In order to determine the optimal MOI and harvest time, four different MOIs of 0.0001, 0.001, 0.01 and 0.1 were tested and compared by virus growth profiling in the cell factories. At the MOI of 0.01, the virus titer reached 10^7.5^ CCID_50_/mL on the fourth day post-infection. At the MOI of 0.001, the virus titer reached 10^6.5^ CCID_50_/mL on the sixth day post-infection (Figure 6). Moreover, the CA16 CC-16 virus induced minimal CPEs in Vero cells at the MOI of 0.01 on the fourth day, which would facilitate the simplification of downstream processing in the production of an inactivated CA16 vaccine. To test whether the temperature could influence the virus growth and yield, the CA16 CC-16 virus was grown in various temperatures from 32 to 37 °C. Finally, the MOI of 0.01, temperature of 35 °C and harvest time of Day 4 post-infection were selected and used in all subsequent experiments.

The culture supernatant of each cell factory was collected in order to avoid large amounts of host cell debris, which would increase difficulties in the downstream purification. Cell debris was removed by filtration through a Zeta Plus filter. To facilitate the downstream purification procedure, the crude virus bulk was concentrated 20- to 30-fold with a 100 KDa cut-off diafiltration membrane in a TFF capsule.

### 3.6. Preliminary Downstream Process and Identification

Chang *et al.* reported employing Sepharose Fast Flow 6 gel and Sephacryl S-500 to purify an EV71 vaccine and obtained similar virus yields and purities with both gel-filtration chromatographies; however, the Sepharose Fast Flow 6 gel was selected because of the commercial availability of its GMP-grade material [30]. Also, taking into consideration the many similarities between EV71 and CA16, we chose the Sepharose Fast Flow 6 gel as the chromatography medium in this study.

**Figure 7:**
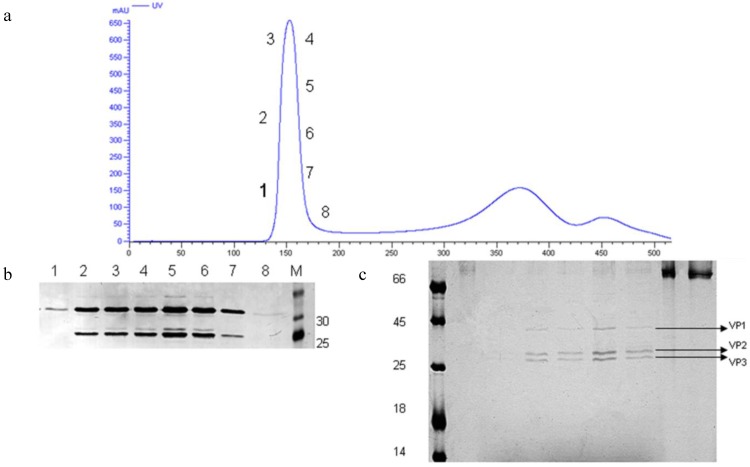
Gel filtration chromatography. (**a**) Elution profile of virus concentrate loaded and separated on the Sepharose Fast Flow 6 column using an AKTA system and monitored by UV absorption at 280 nm. The protein content in each collected fraction was separated and analyzed by Western blot using a CA16 polyclonal antibody (**b**) and silver staining (**c**).

After inactivation with 1:4000 BPL, the concentrated CA16 viral stock was loaded onto the Sepharose Fast Flow 6 gel column and subjected to liquid chromatography. Analysis of each fraction by Western blot using a polyclonal antibody (Figure 7b) and silver staining (Figure 7c) identified the virus mainly in Fractions 2–7. The silver staining indicated that viruses in each fraction were comprised of both solid-core and hollow-core virion. These results were consistent with observations by electron microscopy. Meanwhile, the silver staining indicated that the virus bulk still contained serum and residual host cell proteins after gel-filtration, although these protein levels were very low compared with the viral proteins. The residual host DNA in the CA16 vaccine bulk was determined and found to be <100 pg/mL, which would correspond with the criteria for human vaccine production (100 pg/dose), according to guidelines of the China Food and Drug Administration (sFDA).

### 3.7. Inactivated CA16CC-16 Vaccine Elicits Humoral Immunity in Mice

**Figure 8:**
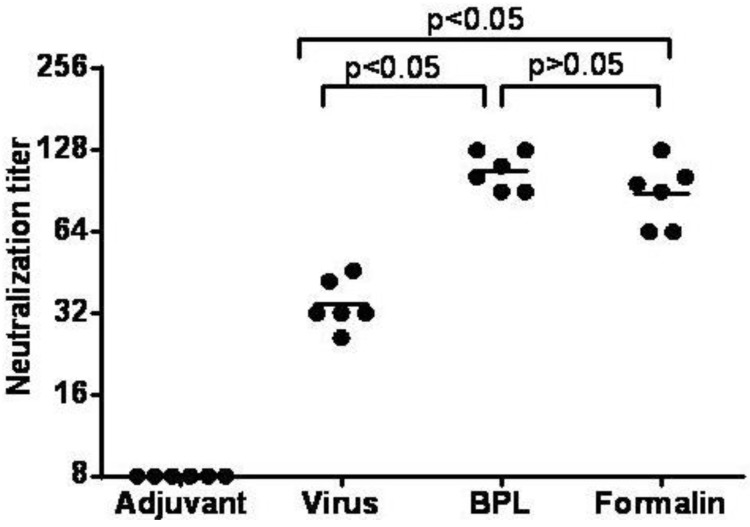
The serum neutralizing antibody responses to CA16 viruses prepared with different inactivation methods. Groups of ICR mice (*n* = 6) were immunized with purified inactivated CA16 with or without adjuvant, or with adjuvant only as a control. Virus represents as inactivated by BPL and without adjuvant, BPL represents as inactivated by BPL and with adjuvant, Formalin represents as inactivated by formalin and with adjuvant Symbols represent reciprocal neutralizing antibody titers from groups of six animals. Neutralization titers (Y-axis) are plotted on a logarithmic scale.

The purified viruses were inactivated by different methods, either with 1/4000 BPL at 4 °C for 72 h or 1/4000 formalin at 37 °C for 120 h, and periodically sampled at 12 h intervals. The virus infectivity was measured using the CPE assay through two rounds of blind passages to determine the time of inactivation. In order to further compare the immunogenicity of different inactivation methods, four groups of mice were immunized using the same procedure with BPL inactivated viruses adsorbed with or without alum adjuvant or formalin inactivated viruses adsorbed with alum adjuvant (only adjuvant group as a negative control). The collected sera were used to analyze the neutralization titer. The antibody geometric mean titers (GMTs) against the CC-16 clone were 23, 107 and 88, respectively (Figure 8). These results showed that the neutralization titers with viruses inactivated with different methods were not significantly different (Figure 8), indicating that the key conformational epitopes of the CA16 virus were not destroyed by formalin or BPL. The BPL inactivated virion without adjuvant formulation elicited the lowest GMT in the four vaccine formulations. The mice receiving BPL inactivated viruses with alum adjuvant formulations had significantly higher neutralizing antibodies than did those who received BPL inactivated viruses with the adjuvant-free vaccine (Figure 8). By comparison, the control group could hardly induce an antibody response. Taking these results together, the CA16 virion inactivated by BPL and with adjuvant formulation was selected as the vaccine for all future studies.

**Figure 9:**
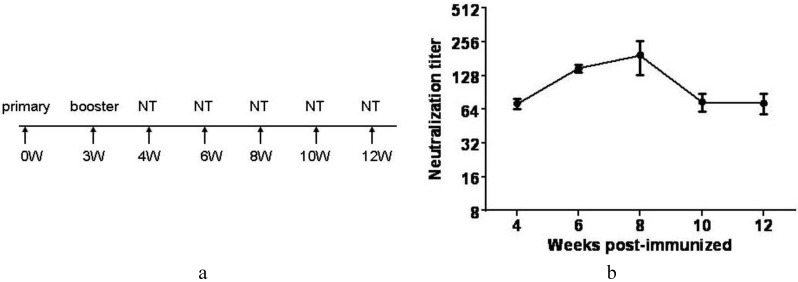
Persistence of CA16 neutralizing antibody. (**a**) Immunization schedule and time points for testing of neutralizing antibody titers; (**b**) CA16 whole virus specific antibody responses were analyzed by measuring neutralizing antibody titers at the 4th, 6th, 8th, 10th and 12th week after primary immunization. Neutralization titers (Y-axis) are plotted on a logarithmic scale.

Furthermore, we collected the serum of immunized mice from the 4th, 6th, 8th, 10th and 12th week, respectively. The neutralizing antibodies against CC-16 manifested the peak activity in the eighth week after primary immunization. Thereafter, it descended rapidly from the 8th to 10th week (Figure 9). These results would potentially help to guide the immunization program in future clinical trials.

## 4. Discussion

CA16 and EV71 both belong to the *Picornaviridae* family of single-stranded positive-sense RNA viruses and can cause HFMD with various neurological symptoms [31]. HFMD has become a serious public health problem in Southeast Asia, with periodic large epidemics occurring in recent decades. Since no effective anti-CA16 agent is available, the development of effective vaccines against CA16 and EV71 infection is the best strategy to prevent and control HFMD.

At present, the whole-virus inactivated EV71 vaccine had completed phase III clinical trials [16] in Mainland China. However, CA16 vaccines are still under development. Based on the successful development and application of the inactivated polio vaccine [32], Wu *et al.* [28] confirmed that a conventional vaccine preparation using whole virus particles currently offers the most expedient method for vaccine development against EV71. In addition, preliminary studies demonstrated that the inactivated EV71 virion elicited more effective immune responses than those by the VP1 protein, DNA vaccine or VLP proteins [28,33]. Therefore, development of an inactivated vaccine appears to be more favorable and feasible compared with the other vaccine types.

Certainly, an ideal inactivated candidate vaccine strain must possess a wide range of cross-neutralizing capacities, strong immunogenicity and genetic stability [20]. According to these standards, in this study, we optimized a CA16 candidate vaccine strain through screening of various strains isolated from cases of HFMD, then we will try to detect the cross neutralization against different subtypes strains. Moreover, we established a neonatal mouse model by utilizing a series of isolated circulating CA16 strains and evaluated the protective efficacy of inactivated CA16 vaccine candidates using this platform. Our study showed that one-day-old neonatal mice born to dams immunized with the experimental inactivated CC024 vaccine showed reduced mortality by 100% after challenge with divergent lethal viruses compared to mice immunized with medium only [22].

A candidate virus with relatively high infective titer and availability of sensitive cell lines are key parameters for inactivated vaccine development. Vero cells as recommended by the World Health Organization (WHO) are used to produce vaccines for viruses such as polio [34] and flu [35,36]. Thus, the ability of all viruses to adapt and proliferate in Vero cells was studied as an important factor for developing an inactivated vaccine, and the CC024 strain revealed a relatively higher titer compared with other strains (Figure 2). Ultimately, the clonal strain CC-16, which was obtained through terminal dilution purification and continuous passaging in Vero cells, showed stable proliferative characteristics with a titer of 10^7.0~7.5^ CCID_50_/mL from Passages 11 to 15. The virus replicated quickly and reached high titers, making it possible to produce a large amount of vaccine in a short period of time to meet immediate demands for HFMD control. Sequence analysis of the virus from the primary, master and working seed banks suggested that the CC-16 strain exhibited genetic stability and stable immunogenicity in mice, which were in line with the requirements of the China Food and Drug Administration. While we have developed techniques for manufacturing of an inactivated CA16 vaccine, further improvements can be made to the upstream process, downstream process and qualification of the vaccine bulk [30]. Currently, we are making great efforts to reduce protein impurities of the virus bulk through various technical methods.

Immunization of mice with the CC024 strain induced high titer antibodies that could neutralize a wide spectrum of CA16 strains *in vitro*. The results suggested that the antibodies produced by CC024 possessed a broad antigenic cross-reactivity and neutralizing ability against all CA16 tested. Meanwhile, these studies provide a research foundation for further CA16 vaccine development and production.

Altogether, results of our studies indicated that the CC-16 clone, as a candidate strain for a CA16 inactivated vaccine, satisfied the requirements of vaccine production in terms of genetic stability, cross-protection and good immunogenicity. The CC-16 candidate strain can be adapted to large-scale production, which would be a desirable feature for vaccine development.

Meanwhile, we demonstrated that the antibodies generated against CA16 could not neutralize EV71 viruses (Figure 1), indicating that an EV71/CA16 bivalent vaccine would be fundamentally necessary to prevent HFMD. Previous research suggested that the inactivated EV71 vaccine would be compatible for co-immunization with another vaccine [37]. Thus, successful development of a whole-virus inactivated CA16 vaccine will contribute to the development and advancement of an EV71/CA16 bivalent vaccine for HFMD.

## 5. Conclusion

In this study we have characterized a CA16CC024 candidate vaccine strain with good genetic stability, high titer in Vero cells and broad-spectrum immunogenicity, according to the guiding principle of the China Food and Drug Administration. Furthermore, preliminary upstream and downstream process were developed for manufacturing of an inactivated CA16 vaccine, various technical methods are feasible. Together, these features indicated that CA16CC024 vaccine strain might have future potential as a CA16 vaccine and EV71/CA16 divalent vaccine.

## Appendix

CC024 GeneBank accession number KF055238

CC045 GeneBank accession number KF055241

CC090 GeneBank accession number KF055243

CC097 GeneBank accession number KF055244

CC163 GeneBank accession number KF055245

G10 GeneBank accession number U05876

SHZH05 GeneBank accession number EU262658
